# Atherosclerotic cardiovascular disease risk stratification and management in type 2 diabetes: review of recent evidence-based guidelines

**DOI:** 10.3389/fcvm.2023.1227769

**Published:** 2023-09-26

**Authors:** Pierre Gourdy, François Schiele, Jean-Michel Halimi, Serge Kownator, Samy Hadjadj, Paul Valensi

**Affiliations:** ^1^ Diabetology Department, Toulouse University Hospital, Toulouse, France; ^2^ Institute of Metabolic and Cardiovascular Diseases, UMR1297 INSERM/UT3, Toulouse University, Toulouse, France; ^3^ Department of Cardiology, University Hospital Besancon, Besancon, France; ^4^ EA3920, University of Franche-Comté, Besancon, France; ^5^ Néphrologie-Immunologie Clinique, Hôpital Bretonneau, CHU Tours, Tours, France; ^6^ EA4245, University of Tours, Tours, France; ^7^ Investigation Network Initiative Cardiovascular and Renal Clinical Trialists (INI-CRCT), Nancy, France; ^8^ Cardiology and Vascular Center, Thionville, France; ^9^ Nantes Université, CHU Nantes, CNRS, INSERM, l’Institut du Thorax, Nantes, France; ^10^ Unit of Endocrinology-Diabetology-Nutrition, AP-HP, Jean Verdier Hospital, Paris 13 University, Sorbonne Paris Cité, CRNH-IdF, CINFO, Bondy, France; ^11^ Polyclinique D'Aubervilliers, Aubervilliers, France

**Keywords:** atherosclerotic cardiovascular disease, type 2 diabetes, risk assessment, risk stratification, guidelines, prevention

## Abstract

Atherosclerotic cardiovascular disease (ASCVD) is the leading cause of mortality and morbidity in individuals with type 2 diabetes mellitus (T2DM). Accordingly, several scientific societies have released clinical practice guidelines to assist health professionals in ASCVD risk management in patients with T2DM. However, some recommendations differ from each other, contributing to uncertainty about the optimal clinical management of patients with T2DM and established ASCVD or at high risk for ASCVD. Thus, the purpose of this paper is to discuss recent evidence-based guidelines on ASCVD risk stratification and prevention in patients with T2DM, in terms of disparities and similarities. To close the gap between different guidelines, a multidisciplinary approach involving general practitioners, endocrinologists, and cardiologists may enhance the coordination of diagnosis, therapy, and long-term follow-up of ASCVD in patients with T2DM.

## Introduction

Atherosclerotic cardiovascular disease (ASCVD), which includes coronary artery disease (CAD), cerebrovascular disease, and peripheral arterial disease (PAD), remains the leading cause of mortality and morbidity in individuals with type 2 diabetes mellitus (T2DM) (1, 2). Among patients with T2DM, prolonged exposure to hyperglycemia and/or insulin resistance were shown to influence several pathophysiological mechanisms that may lead to ASCVD, most notably: the formation of advanced glycation end products; oxidative stress associated with overproduction of reactive oxygen species; protein kinase C activation as a result of increased glucose uptake by vascular cells; and chronic vascular inflammation (3, 4). In addition, the socioeconomic status and built environment of each individual with T2DM may play an important role in the development of ASCVD and its progression (5). Indeed, chronic environmental and psychosocial stressors such as low socioeconomic status, early childhood adversity, social isolation, food insecurity, and decreased sleep quality may lead to chronic inflammation, which in turn can result in ASCVD development and progression (5).

Preventing ASCVD in patients with T2DM should hence be a priority in order to reduce premature death, improve the quality of life, lessen economic burdens, and reduce the risk of chronic kidney disease (CKD) (6). Indeed, considering the close relationship between the metabolic, cardiovascular (CV), and renal systems, not only physiologically but also pathologically, ASCVD and CKD in the presence of T2DM worsen each other (7, 8). Concomitant CKD and T2DM impact the risk for an array of cardiovascular diseases, including arrhythmias, heart failure (HF), acute coronary syndrome, and stroke (9). Prevention of ASCVD in patients with T2DM is thus best addressed through a multifaceted approach including lifestyle changes (i.e., smoking cessation, weight loss, healthy dietary habits, physical activity) and incorporation of specific glucose-lowering medications with cardiovascular and renal benefits, as individually appropriate (2, 9–11). There is, however, large heterogeneity in ASCVD risk among T2DM populations, depending on different factors, including age, sex, diabetes duration, ethnicity, income level, and the presence of traditional risk factors (e.g., smoking, hypertension, dyslipidemia, overweight/obesity) as well as markers of target-organ damage (12, 13). This heterogeneity highlights the need for a personalized approach to ASCVD risk assessment (2, 11, 14).

The present paper discusses recent clinical practice guidelines on ASCVD risk stratification and prevention in patients with T2DM, in terms of disparities and similarities. We conducted a PubMed search coupled with a manual search and focused mainly on guidelines and position papers released in the past 4 years (2019–2023) by various scientific societies, namely, the European Society of Cardiology (ESC)/European Association for the Study of Diabetes (EASD) (10), the American Diabetes Association (ADA) (2), the ADA/EASD (11), the American College of Cardiology (ACC)/American Heart Association (AHA) (12, 15), the American Association of Clinical Endocrinologists (AACE)/American College of Endocrinology (ACE) (16, 17), Diabetes Canada (18), the French Society of Cardiology (SFC)/French-Speaking Society of Diabetology (SFD) (13), and the United Kingdom National Institute for Health and Care Excellence (NICE) (19).

## How is ASCVD risk initially stratified according to the clinical profile of patients with T2DM?

There are some disparities and varying approaches adopted by different clinical practice guidelines regarding initial ASCVD risk stratification in patients with T2DM.

Both the 2019 ESC/EASD joint guidelines on diabetes, pre-diabetes, and cardiovascular diseases and the SFC/SFD's position paper (13) do not recommend assessing ASCVD risk in patients with T2DM based on global risk scores derived in the general population (10). They instead categorize patients with T2DM as being at moderate, high, or very high risk for ASCVD depending on diabetes duration, presence of target-organ damage, and of concomitant risk factors (Table 1). The 2020 AACE/ACE consensus statement on the management of dyslipidemia and prevention of cardiovascular disease (16), however, considers T2DM as an ASCVD risk equivalent. Individuals with T2DM are, therefore, classified by the AACE/ACE as being at high, very high, or extreme ASCVD risk (Table 1).

**Table 1:** ASCVD risk categories applying to patients with T2DM according to different guidelines.

ESC/EASD (10)		AACE/ACE (16)		SFC/SFD (13)	
Very high risk (10-year risk of CV death >10%)	Patients with T2DM and established ASCVD or target-organ damage (proteinuria, renal impairment defined as eGFR <30 ml/min/1.73 m^2^, left ventricular hypertrophy, or retinopathy) or ≥3 major risk factors (age >65 years, hypertension, dyslipidemia, smoking, obesity, microalbuminuria)	Extreme risk (10-year risk of 3-point MACE^a^ >30%)	Patients with established clinical ASCVD + T2DM	Very high risk	Patients with T2DM aged 35–75 years with ≥1 of the following: - Previous CV disease - LDL cholesterol >190 mg/dl despite treatment - Albuminuria >300 mg/24 h or 200 mg/L or albumin/creatinine ratio on spot urine ≥300 mg/g - eGFR <30 ml/min/1.73 m^2^ - Abnormal Q waves on ECG - Abnormal left ventricular function or hypertrophy on echocardiography - Peripheral artery atheromatous stenosis ≥50%
High risk (10-year risk of CV death of 5%–10%)	Patients with T2DM duration ≥10 years without target-organ damage, plus any other additional risk factor	Very high risk (10-year 3-point MACE^a^ risk >20%–30%**)**	Patients with T2DM with ≥1 risk factor(s) including advancing age, hypertension, cigarette smoking, CKD, family history of ASCVD, dyslipidemia, obesity	High risk	Patients with T2DM aged 35–75 years with ≥2 of the following: - T2DM duration ≥10 years - Premature CAD in a first-degree relative (men <50 years; women <60 years) - Persistently uncontrolled risk factors (HbA1c, LDL, non-HDL cholesterol, blood pressure, smoking) - Confirmed albuminuria (30–300 mg/24 h or 20–200 mg/L or eGFR 30–60 ml/min/1.73 m^2^) - Severe retinopathy or autonomic neuropathy or erectile dysfunction - Low physical activity
Moderate risk (10-year risk of CV death <5%)	Patients aged <50 years with T2DM duration <10 years, with no other risk factors	High risk (10-year 3-point MACE^a^ risk of 10%–20%)	Patients with T2DM with no other risk factors	Moderate risk	Patients who are not at high or very high risk

HDL, high-density lipoprotein; LDL, low-density lipoprotein.

^a^ 3-point MACE is defined as a composite of non-fatal stroke, non-fatal myocardial infarction, and cardiovascular death.

By contrast, both the ADA (2) and ACC/AHA clinical practice guidelines on the management of blood cholesterol in people with diabetes (12) recommend a global estimation of ASCVD risk in patients with T2DM, using the ACC/AHA ASCVD Risk Estimator Plus (Table 2). Although the ASCVD Risk Estimator Plus includes diabetes as a risk factor, it does not account for type of diabetes (type 1 or 2), diabetes duration, or the presence of diabetes complications such as albuminuria (2). Of note, in a multiethnic, large, real-world population, the ACC/AHA risk score was found to overestimate the risk of ASCVD in adults with or without diabetes (20, 21).

**Table 2:** Features of different risk assessment tools.

Risk assessment tool	Included variables	Predicted outcomes	10-year ASCVD risk stratification
ASCVD risk estimator plus (https://tools.acc.org/ASCVD-Risk-Estimator-Plus/#!/calculate/estimate/): designed for the general population	Age Sex Race Systolic blood pressure Diastolic blood pressure Total cholesterol HDL cholesterol LDL cholesterol History of diabetes Smoking status On antihypertensive therapy On statins On aspirin therapy	Hard ASCVD (death from CAD, non-fatal MI, fatal or non-fatal stroke)	Low risk (<5%) Borderline risk (5%–7.4%) Intermediate risk (7.5%–19.9%) High risk (≥20%)
QRISK2 score (https://www.qrisk.org/2017/): designed for the general population	Age Sex Ethnicity Smoking status Diabetes status Angina or MI in a first-degree relative <60 years CKD stage 4 or 5 Presence of atrial fibrillation On antihypertensive therapy Rheumatoid arthritis Cholesterol/HDL ratio Systolic blood pressure Body mass index	Hard ASCVD (10-year risk of MI or stroke)	Low risk (<10%) Moderate risk (10%–20%) High risk (>20%)
UKPDS risk engine (https://www.dtu.ox.ac.uk/riskengine/): designed for patients with T2DM	Age Sex Ethnicity Smoking status Presence of atrial fibrillation T2DM duration HbA1c level Systolic blood pressure Total cholesterol HDL cholesterol	- CAD - Fatal CAD - Stroke - Fatal stroke	Low risk (<15%) Medium risk (15%–29.9%) High risk (≥30%)
ADVANCE risk score (https://u-prevent.com/calculators/advanceScore): Designed for patients with T2DM	Age Sex Current smoking Presence of atrial fibrillation T2DM duration Presence of retinopathy HbA1c level Treated hypertension Systolic blood pressure Diastolic blood pressure Total cholesterol HDL cholesterol LDL cholesterol Albumin-to-creatinine ratio	Hard ASCVD (10-year risk of MI, stroke, or cardiovascular death)	Not reported

The current NICE guidelines (19) recommend the use of QRISK2 (Table 2). The NICE considers that adults with T2DM aged ≥40 years with a QRISK2 score >10% should be classified as having a high risk of developing cardiovascular disease. However, given that QRISK2 does not estimate lifetime cardiovascular risk, the NICE also takes into account additional risk factors (i.e., hypertension, smoking, dyslipidemia, obesity, family history of premature cardiovascular disease in a first-degree relative) and considers that people aged under 40 years with T2DM and at least one cardiovascular risk factor have an elevated lifetime risk of ASCVD (19). Of note, the ASCVD risk calculated by QRISK2 is likely to be underestimated in individuals from deprived areas and overestimated for those from affluent areas (22).

A T2DM-specific risk calculator has been suggested by the AACE/ACE (16), based on the UK Prospective Diabetes Study (UKPDS) Risk Engine (Table 2). However, using prospective data from 1,482 Dutch men and women aged 50–75 years, it was revealed that the UKPDS risk model has a low discriminatory ability among patients with T2DM for estimating the risk of the first CAD event, and a moderate ability to identify individuals with a high risk for a fatal CAD event (23).

Finally, the European Association of Preventive Cardiology (EAPC) (14) advises the use of the U-Prevent tool (www.U-Prevent.com), which is an interactive website incorporating ASCVD risk calculators for different categories of patients, including the ADVANCE risk score for patients with T2DM. The ADVANCE risk score is also available via the free “ESC CVD Risk Calculation” app. The ADVANCE risk score (24) takes into consideration diabetes-specific variables, such as diabetes duration; glycated hemoglobin (HbA1c); the presence of retinopathy, albuminuria, and atrial fibrillation; in addition to classic risk factors (Table 2), in order to provide the 4-year risk of myocardial infarction (MI), stroke, or cardiovascular death (14). To facilitate clinical interpretation, the U-Prevent tool extrapolates the 4-year risk of ASCVD as estimated by the ADVANCE risk score to 10 years. The ADVANCE risk score has been successfully validated in contemporary real-world populations from various countries (14, 24, 25).

It is overall more accurate to adopt an ASCVD risk stratification model that considers the presence of several risk factors when classifying patients with T2DM as having a very high ASCVD risk*.* In the Swedish National Diabetes Register (26), a nationwide cohort study, including 271,174 patients with T2DM matched with controls, patients were evaluated according to the presence of five traditional risk factors [HbA1c ≥7.0% (53 mmol/mol), low-density lipoprotein cholesterol ≥2.5 mmol/L (97 mg/dl), albuminuria, smoking, and blood pressure ≥140/80 mmHg]. It was found that patients with T2DM who had five risk factor variables within recommended target ranges had little or no excess risks of death, MI, and stroke when compared with the general population (26). These findings highlight that even if multiple traditional risk factors are present, their control can substantially reduce the risk of cardiovascular events among patients with T2DM (26).

Regardless of a patient's initial ASCVD risk score, risk-enhancing factors as well as additional testing to assess the presence of subclinical atherosclerosis may reclassify ASCVD risk and overcome the potential imprecision of standard ASCVD risk calculators (16).

## How should further ASCVD risk assessment be undertaken in patients with T2DM?

Beyond traditional risk factors that have been incorporated into the standard ASCVD risk calculators, clinical practice guidelines (10, 12, 13, 16) support assessing additional risk factors or markers, dubbed “risk enhancers” or “risk modifiers,” which may significantly alter ASCVD risk in subsets of patients with T2DM and better predict future ASCVD events (Table 3).

**Table 3:** Risk-enhancing factors according to ACC/AHA (12).

Specific to diabetes	In the general population
T2DM duration ≥10 years	Family history of premature ASCVD
Albuminuria ≥30 µg of albumin/mg creatinine	LDL cholesterol ≥160 mg/dl
eGFR <60 ml/min/1.73 m^2^	Metabolic syndrome^a^
Retinopathy	Chronic kidney disease
Neuropathy	History of preeclampsia or premature menopause in women
Ankle-brachial index <0.90	Chronic inflammatory disorders
	High-risk ethnicity such as South Asian ancestry
	Triglyceride levels persistently >175 mg/dl
	If measured: - Apolipoprotein B levels with elevations >130 mg/dl (may be useful if hypertriglyceridemia >200 mg/dl to rule out genetic disorders such as type III hyperlipoproteinemia or to clarify ASCVD risk) - High-sensitivity C-reactive protein ≥2 mg/L - Lipoprotein(a) levels with elevations >50 mg/dl (>125 nmol/L). Elevated lipoprotein(a) levels may be especially useful in those with a family history of ASCVD - Ankle-brachial index <0.90

^a^ Defined as a cluster of cardiovascular and diabetes risk factors including abdominal obesity, dyslipidemia, glucose intolerance, and hypertension (27).

Various tests have also been proposed by clinical practice guidelines to measure subclinical atherosclerosis and optimize ASCVD risk assessment in individuals with diabetes. According to the AACE/ACE (16), these tests may include resting electrocardiogram (ECG), stress tests [i.e., exercise and/or pharmacologic stress tests, stress imaging (single-photon emission computed tomography, echocardiography, or cardiac magnetic resonance imaging)], ankle-brachial index (ABI), coronary artery calcium (CAC) testing, and carotid/femoral ultrasound.

All clinical practice guidelines uniformly endorse the use of resting ECG for routine evaluation of asymptomatic patients with diabetes (2, 10, 13, 16). Of note, approximately 60% of MIs in patients with T2DM may be asymptomatic, thus only discovered by resting ECG (28). Any significant abnormality on resting ECG should lead to stress ECG and/or echocardiography (2).

Measurement of the ABI has also been recommended to be performed in all patients with T2DM by the ESC/EASD (10), the SFC/SFD (13), and the ACC/AHA (12). An ABI value <0.90 is diagnostic for PAD, and both ABI values <0.90 and >1.40 are associated with an increased risk of cardiovascular events and death (10, 13). However, given that high ABI values may indicate the presence of medial arterial calcification, a characteristic ultrasound feature of T2DM, the 2019 ESC/EASD guidelines (10) recommend other non-invasive tests, such as the toe-brachial index or duplex ultrasound, in patients with an ABI >1.40.

By assessing the volume of coronary calcifications and assuming that each calcification represents an atherosclerotic plaque, CAC testing may further improve ASCVD risk prediction in people with T2DM and allow patient reclassification. A CAC score ≤10 indicates a very low 10-year risk of cardiovascular events, 11–100 a low risk, 101–400 a moderate risk, and >400 a high risk (13, 29). Individuals with a CAC score >100 and, in particular, a CAC score >400 may be good candidates for aspirin therapy for primary cardiovascular prevention (30). Conversely, a CAC score of 0 is a strong negative risk marker of ASCVD events. In the general population, a CAC score of 0 confers “a warranty period against mortality” of 15 years, which is reduced to 5 years in patients with T2DM (31). Moreover, a CAC score of 0 that persists beyond 5 years is strongly associated with a low risk of ASCVD events among patients with T2DM (32).

Of note, since the CAC score has been found to be highly related to age, it is important to take age into account to better discriminate between lower and higher CAD risk (33). For example, the SFC/SFD specifies that a patient aged ≥60 years with a CAC score of 101–400 should be categorized as having a high risk of cardiovascular events, whereas a patient aged <60 years with the same CAC score would be categorized as having a very high risk (13). So far, the Multi-Ethnic Study of Atherosclerosis (MESA) risk calculator (https://www.mesa-nhlbi.org/MESACHDRisk/MesaRiskScore/RiskScore.aspx) is the only available algorithm incorporating the CAC score with traditional risk factors such as diabetes for the 10-year CAD risk prediction.

However, the ADA (2) and the ACC/AHA guidelines (12) do not recommend the routine use of CAD screening methods, including CAC testing, in risk stratification of patients with T2DM. The ADA recommends investigations for CAD using CAC testing, only in the presence of an abnormal ECG (e.g., Q waves), atypical cardiac symptoms (e.g., chest discomfort, unexplained dyspnea), or signs/symptoms of associated vascular disease including carotid bruits, transient ischemic attack (TIA), stroke, claudication, or PAD (2). The ACC/AHA recommendation (12) against routine CAC testing is based on a large population-based study of adults with T2DM and without ASCVD who had a CAC score of 0, but for whom a mean 10-year ASCVD risk of 8.0% was found, indicating that they were not at low risk of cardiovascular events (34).

By contrast, the 2019 ESC/EASD guidelines (10) and SFC/SFD (13) recommend CAC testing to improve cardiovascular risk stratification, as patients with diabetes are a heterogeneous population. Since high CAC scores are associated with an increased risk of silent CAD in patients with T2DM (35), SFC/SFD (13) support CAC testing in patients with T2DM *a priori* considered to be at high ASCVD risk, based on T2DM duration (≥10 years), the presence of microvascular complications (i.e., microalbuminuria, severe retinopathy, autonomic neuropathy, or erectile dysfunction), family history of premature ASCVD, and persistently uncontrolled risk factors (i.e., hyperglycemia, hypertension, smoking, dyslipidemia). In case of a CAC score >400, both the ESC/EASD (10) and SFC/SFD (13) recommendations endorse further cardiac investigations using coronary computed tomography angiography or functional imaging (e.g., stress echocardiography, radionuclide myocardial perfusion imaging, stress cardiac magnetic resonance imaging).

Beyond CAC testing, carotid ultrasound can give information on both intimal-media thickness (IMT) and the presence and characteristics of atherosclerotic plaques. However, several clinical practice guidelines do not recommend carotid ultrasound IMT for ASCVD risk assessment due to its weak specificity and reproducibility (10, 13, 16). Moreover, in patients with T2DM, carotid IMT does not have an incremental value over the CAC score for predicting CAD or cardiovascular events (34).

Although the routine use of resting ECG in evaluating patients with T2DM and suspected ASCVD is widely agreed upon, there are still many knowledge gaps on the prognostic value of the different tests used in ASCVD risk assessment, which may partially explain the discrepancies that emerge from different clinical practice guidelines. In addition, clinical practice guidelines do not provide clear recommendations on how frequently to re-evaluate ASCVD risk in patients with T2DM. The distinction between primary and secondary prevention of ASCVD has also become blurrier because imaging techniques such as CAC testing and carotid/femoral plaque imaging may reveal subclinical atherosclerosis in asymptomatic patients with T2DM (29). The results of a recent retrospective study, evaluating the validity of the 2019 ESC/EASD guidelines for cardiovascular risk stratification (10), support CAC testing in patients with T2DM without known ASCVD (36). CAC testing may hence be considered a non-invasive, cost-effective, and quick technique that can provide a substantial improvement in risk reclassification and may justify further investigations to assess patients for silent CAD or ischemia (36). The recommended time for repeat CAC testing is approximately 3 years in individuals with T2DM (37). Similarly, since the ABI enables risk reclassification, a comprehensive peripheral vascular evaluation including ABI measurement could be considered in all patients with T2DM, knowing that PAD remains frequently underdiagnosed and undertreated, and patients with T2DM are more prone to PAD than the general population. In general, ABI measurement should be performed annually in patients with T2DM (38).

## How should appropriate glucose lowering agents be chosen for patients with or at a high risk for ASCVD?

Recommendations from different clinical practice guidelines are overall consistent regarding the therapeutic management of T2DM in patients with or at a high risk for ASCVD. All clinical practice guidelines endorse a comprehensive approach to the management of cardiovascular risk factors in patients with T2DM, including hyperglycemia, high blood pressure, dyslipidemia, obesity, smoking, and thrombotic risk, using a patient-centered approach (2, 9–11, 13, 15, 17, 19). Weight loss in particular can influence ASCVD risk in patients with T2DM, especially those with obesity. Indeed, weight loss can lead to a cascade of positive effects, including improved insulin sensitivity, decreased inflammation, improved lipid profile, and blood pressure reduction. These combined effects contribute to an overall reduction in the risk of ASCVD and mortality in T2DM (9).

The combined reduction of HbA1c [with a target <7.0% (53 mmol/mol) for most adults], blood pressure, and lipids can decrease the risk of microvascular and macrovascular events by around 50% and hospitalization for heart failure (HHF) by 70% (39, 40). Multifactorial treatment remains, however, underused and/or fails to achieve optimal targets for all cardiovascular risk factors (10). In a real-world cohort study from the United States of 324,706 patients with T2DM and ASCVD, a statin was prescribed to 58.6% of patients, an angiotensin-converting enzyme inhibitor or an angiotensin-receptor blocker to 45.5%, and only 6.7% of patients were prescribed a cardioprotective glucose-lowering agent such as glucagon-like peptide-1 receptor agonists (GLP-1RAs) or sodium-glucose cotransporter-2 inhibitors (SGLT2is) (41).

Based on guidelines from the ESC/EASD (10), the ADA (2), and the ADA/EASD (11), GLP-1RAs or SGLT2is should be considered as first-line therapy in individuals with high ASCVD risk or established ASCVD, as their cardiovascular benefits are thought to be largely independent of their glucose-lowering properties. Guidelines from the ACC/AHA (15), the AACE/ACE (17), and Diabetes Canada (18), however, recommend metformin as first-line therapy, with GLP-1RAs or SGLT2is administered as an adjunct to metformin in patients with or at high risk for cardiovascular events. On their part, the NICE (19) only recommends as first-line therapy SGLT2is in addition to metformin in patients with T2DM and high ASCVD risk/established ASCVD or chronic HF. For the NICE, GLP-1RAs are not cost-effective as a class (19).

According to the 2023 ADA clinical practice guidelines (2), adoption of GLP-1RAs or SGLT2is should be straightforward in patients with newly diagnosed T2DM and high ASCVD risk or established ASCVD, as these cardioprotective agents can be used from the beginning of diabetes management. However, the incorporation of GLP-1RA or sodium-glucose cotransporter-2 inhibitor (SGLT2i) therapy in the care of patients with long-standing T2DM may be more challenging, especially if patients are using an already complex glucose-lowering regimen. In such patients, GLP-1RA or SGLT2i therapy may need to replace some or all of their existing medications to minimize adverse side effects and hypoglycemia risk, as well as medication costs (2). The NICE (19) proposes that when starting a patient with T2DM on dual therapy with metformin and SGLT2i, the drugs should be introduced sequentially, starting with metformin and assessing tolerability. As soon as metformin tolerability is confirmed, the SGLT2i can then be started to reduce clinical inertia and optimize cardiovascular benefit (19).

Large observational studies and meta-analyses of large-scale cardiovascular outcome trials (CVOTs) have both shown that the two drug classes reduce the risks of MI and cardiovascular death to a comparable extent in patients with T2DM (42, 43). Based on the results of CVOTs, the most reliable evidence for a benefit on major adverse cardiovascular events (MACE) (comprising MI, stroke, and all-cause mortality) is for liraglutide, dulaglutide, and semaglutide among GLP-1RAs, and empagliflozin, canagliflozin, and dapagliflozin among SGLT2is (2, 9–11, 13, 18, 19). Hence, the use of either drug class to reduce the risk of MACE in patients with T2DM and established ASCVD is appropriate, considering the contraindications and side effects of each of the two classes of drugs (2). According to Diabetes Canada (18), the strongest evidence for a cardiovascular benefit in patients with T2DM without established ASCVD comes from the REWIND (Researching cardiovascular Events with a Weekly INcretin in Diabetes) CVOT, evaluating dulaglutide in 9,901 participants with T2DM, 69% of whom had no previous ASCVD but had either renal impairment or ≥2 cardiovascular risk factors (abdominal obesity, hypertension, smoking, or dyslipidemia) (44). The effects of SGLT2is on MACE in people with T2DM without preexisting ASCVD remain unclear (18).

Compared to placebo, SGLT2is, namely empagliflozin, canagliflozin, and dapagliflozin, have been found to reduce the risk of HHF by 27%–35% in CVOTs of patients with T2DM with and without established ASCVD (45–47). Accordingly, various clinical practice guidelines recommend the use of SGLT2is to decrease the risk of HHF in patients with T2DM and established ASCVD or multiple ASCVD risk factors (2, 9–11, 13, 18). According to the latest guidelines by the ADA, the EASD, and the ESC (2, 11, 48), SGLT2is should be prioritized in patients with T2DM and established HF, particularly those with a reduced ejection fraction (HFrEF), to reduce the risk of worsening HF and cardiovascular death. This recommendation was mainly based on results from the EMPEROR-Reduced (EMPagliflozin outcomE tRial in patients with chrOnic heaRt failure with Reduced Ejection Fraction) (49) and DAPA-HF (Dapagliflozin And Prevention of Adverse-outcomes in Heart Failure) (50) trials. In these two CVOTs conducted in patients with HFrEF, empagliflozin and dapagliflozin were both associated with significantly lower risks of cardiovascular death and HHF compared to placebo, regardless of the absence or presence of T2DM (49, 50). Similar results were published with empagliflozin and dapagliflozin in patients with HF and a preserved ejection fraction (51–53).

SGLT2is are also recommended by the AACE/ACE (17), the ADA (2), the ESC/EASD (10), Diabetes Canada (18), the ADA/EASD (11), and Kidney Disease: Improving Global Outcomes (KDIGO) (54) to reduce nephropathy progression among patients with T2DM and established ASCVD, multiple risk factors for ASCVD, or CKD, as long as the estimated glomerular filtration rate (eGFR) is ≥20 ml/min/1.73 m^2^. Three SGLT2is are recommended for nephroprotection in patients with T2DM, namely, canagliflozin, dapagliflozin, and empagliflozin, based on the results of the CREDENCE (Canagliflozin and Renal Events in Diabetes with Established Nephropathy Clinical Evaluation) (55), DAPA-CKD (Dapagliflozin and Prevention of Adverse Outcomes in Chronic Kidney Disease) (56), EMPA-REG OUTCOME (Empagliflozin Cardiovascular Outcome Event Trial in Type 2 Diabetes Mellitus Patients) (57), and EMPA-KIDNEY (The Study of Heart and Kidney Protection With Empagliflozin) (58) trials, respectively. In a meta-analysis of 13 CVOTs, SGLT2is were found to safely lower the risk of CKD progression, acute kidney injury, cardiovascular death, and HHF in patients with CKD or HF, irrespective of the level of kidney function or diabetes status (59).

GLP-1RAs have also shown to decrease albuminuria and slow eGFR decline, although to a lesser extent than SGLT2is (54). In a meta-analysis of six CVOTs performed in patients with T2DM, GLP-1RAs significantly reduced the risk of a composite kidney disease outcome (macroalbuminuria, doubling of serum creatinine or eGFR decline, kidney replacement therapy, or death from kidney disease) compared with placebo by 21% (60). Accordingly, the 2022 KDIGO guidelines recommend GLP-1RA use in patients with T2DM and CKD who did not achieve individualized glycemic targets despite metformin use and SGLT2i treatment, or who are unable to use those medications (54). Thus, both SGLT2is and GLP-1RAs represent critical advancements in delaying the progression of CKD in T2DM, which is traditionally managed by glycemic control, blood pressure control, and renin-angiotensin-aldosterone system inhibition (9). Another promising treatment option to delay CKD progression in patients with T2DM is finerenone, a non-steroidal mineralocorticoid receptor antagonist (61). In the FIGARO-DKD (Finerenone in Reducing Cardiovascular Mortality and Morbidity in Diabetic Kidney Disease) trial performed in patients with T2DM and CKD, compared with placebo, finerenone improved cardiovascular outcomes and reduced the risk of the kidney composite outcome of kidney failure, a sustained ≥57% decrease in eGFR from baseline, or renal death (61).

It has been pointed out by the SFC/SFD (13) and by Diabetes Canada (18) that both GLP-1RAs and SGLT2is confer a low hypoglycemia risk and with a weight loss effect compared to other glucose-lowering agents such as insulin or sulfonylureas. Thus, Diabetes Canada (18) also proposes the use of GLP-1RAs and/or SGLT2is in patients with T2DM with or without established ASCVD for whom reducing hypoglycemia risk and/or inducing weight loss (i.e., in case of obesity) are priorities. Of note, in the SURMOUNT-1 phase III trial performed in adults with obesity, once-weekly tirzepatide, a dual agonist of GLP-1 and glucose-dependent insulinotropic polypeptide (GIP), demonstrated substantial reductions in body weight of up to 22.5%, with greater improvements than placebo in cardiometabolic risk factors, including waist circumference, systolic and diastolic blood pressure, and fasting insulin, lipid, and aspartate aminotransferase levels (62).

In the absence of direct comparisons and depending on the presence or absence of HF and CKD, data from CVOTs can provide some guidance for clinicians choosing in a patient-centered manner between these two classes of drugs in patients with T2DM and established ASCVD or multiple risk factors for ASCVD (43). Hence, in patients with a history of stroke/TIA as well as in patients with prior PAD, the use of GLP-1RAs over SGLT2is may be preferred. By contrast, in patients with CKD and/or a history of HF, the use of SGLT2is over GLP-1RAs may be preferred (43). It is important to note that although the cardiovascular benefits of GLP-1RAs and SGLT2is are firmly established for individuals with T2DM and known ASCVD, there is less evidence among those with T2DM and without established ASCVD regarding the beneficial effects of GLP-1RAs and SGLT2is on MACE in primary cardiovascular prevention, whereas the benefit of SGLT2is on HHF is consistent irrespective of the absence or presence of ASCVD in a patient (9, 43).

The ESC/EASD and the ADA recommend considering sequential GLP-1RA and SGLT2i combination therapy for additive reduction in the risk of adverse cardiorenal events among patients with T2DM and established ASCVD or at high risk for ASCVD (2, 10, 11). The ESC/EASD (10) justifies GLP-1RA and SGLT2i combination therapy by the different mechanistic effects of these two drug classes, as the cardiovascular benefits of SGLT2is are primarily due to their hemodynamic actions, while those of GLP-1RAs are mainly due to their anti-atherogenic effects. On its part, the ADA (2) justifies GLP-1RA and SGLT2i combination therapy based on findings from the AMPLITUDE-O (Effect of Efpeglenatide on Cardiovascular Outcomes) CVOT (63, 64), which evaluated the investigational GLP-1RA efpeglenatide in 4,076 patients with T2DM and either a history of cardiovascular disease or current kidney disease, 618 (15.2%) of whom reported SGLT2i use at baseline. Over a median follow-up of 1.8 years, efpeglenatide therapy reduced the risk of MACE by 27% and of a composite renal outcome by 32%. Importantly, the efficacy and safety of efpeglenatide did not differ by SGLT2i use, suggesting that the beneficial effects of the GLP-1RA were independent of those provided by SGLT2i therapy (64). However, there are some potential challenges in real-world practice regarding GLP-1RA and SGLT2i combination therapy that should be further explored, including the impact of the frequency of administration and the different routes of administration (subcutaneous for almost all GLP-1RAs and oral for SGLT2is) on patient adherence, as well as cost-effectiveness of combination therapy (65, 66).

Of note, all GLP-1RA and SGLT2i CVOTs have been conducted in patients with long-standing T2DM who were already treated with a glucose-lowering regimen. There is, therefore, no clinical evidence for the cardiovascular benefits of GLP-1RAs and SGLT2is in patients with newly diagnosed T2DM. However, based on the current cumulative evidence, clinical practice guidelines from the ESC/EASD (10), ADA (2), and Diabetes Canada (18) recommend GLP-1RAs and/or SGLT2is in newly diagnosed patients with T2DM and with ASCVD or multiple risk factors for ASCVD.

## Conclusion

**Figure 1:**
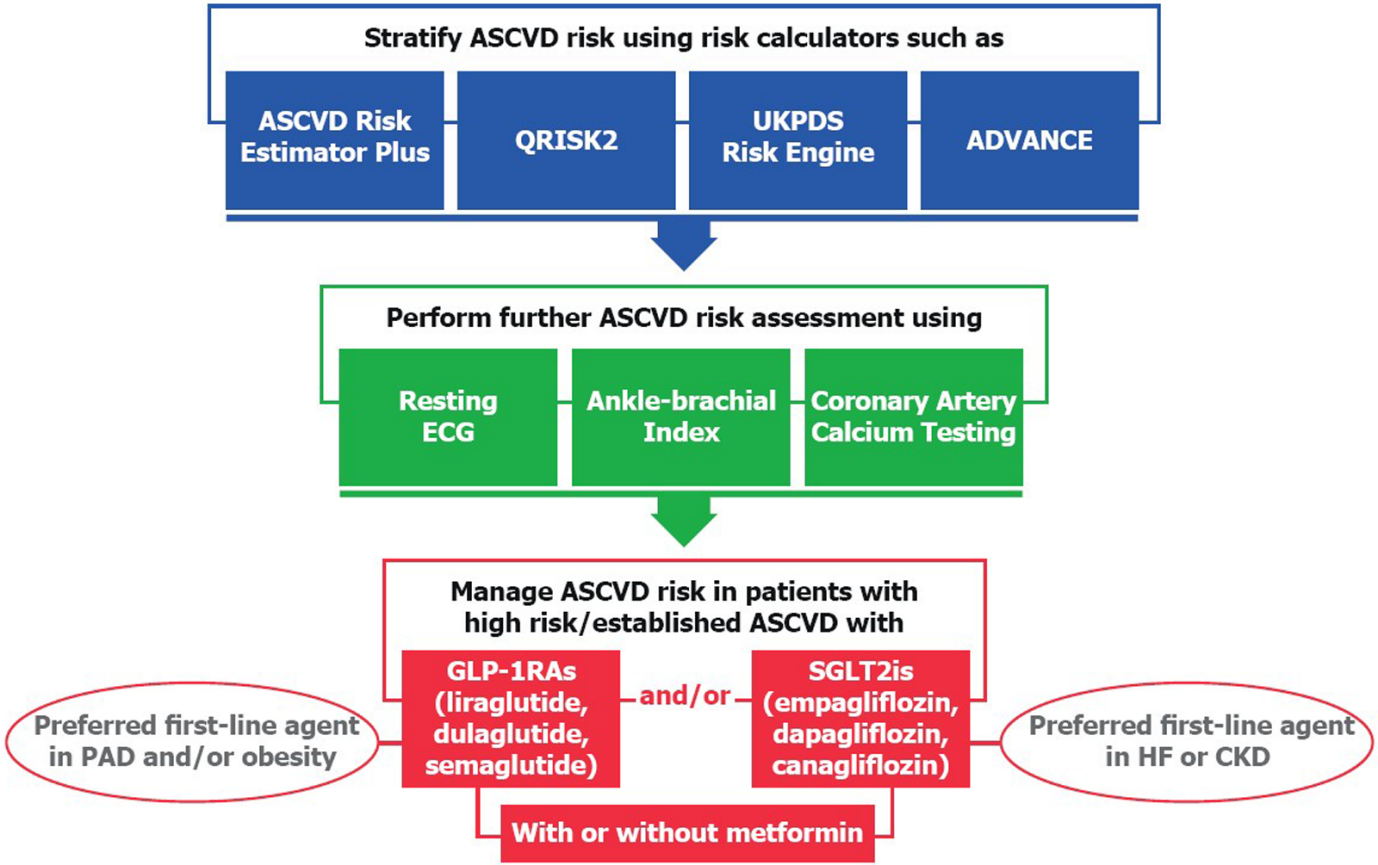
A summary of evidence-based guidelines on ASCVD risk management in type 2 diabetes.

Several scientific societies have released clinical practice guidelines to assist health professionals in ASCVD risk stratification and prevention in patients with T2DM. Figure 1 summarizes ASCVD risk management in patients with T2DM according to evidence-based guidelines (2, 9–11, 13, 15, 17, 19). To reduce the risk of ASCVD among patients with T2DM, clinical practice guidelines consistently recommend the use of SGLT2is and/or GLP-1RAs, which have shown both cardiovascular and renal protective effects, thus supporting the idea of a multidirectional relationship between T2DM, ASCVD, and CKD, which may partially explain benefits with these glucose-lowering agents across different organ systems. There are, however, disparities between different guidelines that create a challenging gap between evidence generation and real-world clinical practice for patients with T2DM with high ASCVD risk or established ASCVD. To close this gap, we encourage a multidisciplinary approach involving general practitioners, endocrinologists, and cardiologists to coordinate diagnosis, therapy, and long-term follow-up (67). These specialists are invited to embrace a comprehensive, individualized ASCVD risk assessment and management. Efforts should also be made regarding continuous patient education and support, which are necessary to improve disease knowledge and management as well as patient empowerment. To raise the standards of preventive cardiology in patients with T2DM, several issues remain to be explored, ranging from ASCVD risk management in older people to the comparative efficacy and safety of GLP-1RAs vs. SGLT2is as well as combination of both drug classes.
